# Supplementary material for: Cell-Free Supernatant from Lactobacillus and Streptococcus Strains Modulate Mucus Production via Nf-κB/CREB Pathway in Diesel Particle Matter-Stimulated NCI-H292 Airway Epithelial Cells
Source: Molecules. 2022 Dec 21;28(1):61. doi: 10.3390/molecules28010061 (PMC9822189; doi:10.3390/molecules28010061)
Supplement: Supplementary file 1 [file molecules-28-00061-s001.zip › molecules-1985099-supplementary.pdf]

**Table S1.** Primer sequence for qRT-PCR amplification in RAW 264.7 cells.

| Gene † | | Sequence (5' - 3') | Product (bp) | Annealing temperature (°C) |
|---|---|---|---|---|
| *IL6* | Forward | TACCACTTCACAAGTCGGAGGC | 116 | 59 |
| | Reverse | CTGCAAGTGCATCATCGTTGTTC | | |
| *TNFα* | Forward | GGTGCCTATGTCTCAGCCTCTT | 139 | 59 |
| | Reverse | GCCATAGAACTGATGAGAGGGAG | | |
| *GADPH* | Forward | TCTCCCTCACAATTTCCATCC | 100 | 59 |
| | Reverse | GGGTGCAGCGAACTTTATTG | | |

† *IL6*, interleukin 6; *TNF-α*, tumor necrosis factor alpha; *GAPDH*, glyceraldehyde 3-phosphate dehydrogenase.

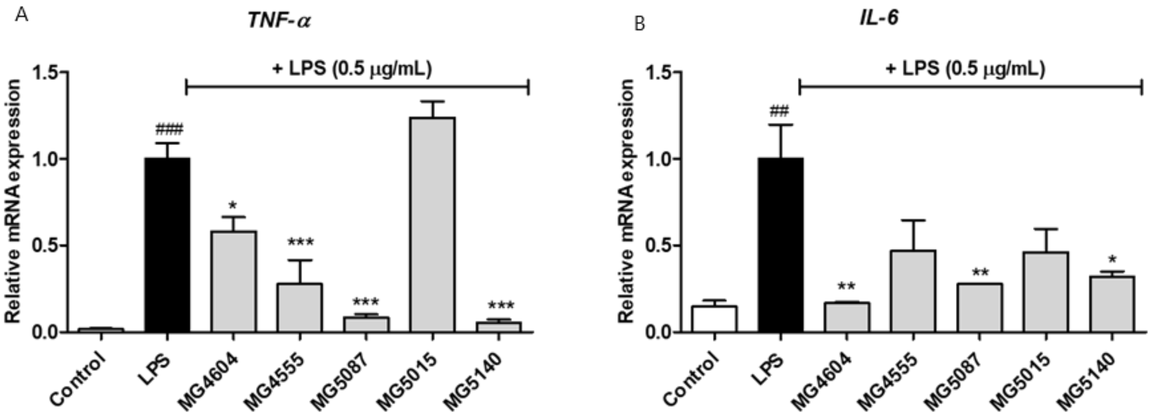


**Figure S1.** Effect of CFS from *Lactobacillus* and *Streptococcus* strains (except MG4272, MG4577, and MG4247) on *TNF-α* (**A**), and *IL-6* (**B**) mRNA expression in LPS-induced RAW264.7 cells. The expression of mRNA was determined by qRT-PCR. The RAW264.7 cells were pretreated with 5% of CFS and then incubated with or only LPS (0.5 μg/mL) for 3 h. The mRNA expression was normalized to *GAPDH* as the internal control. The results indicate the mean ± SEM of four separate experiments. $^{\#\#}p < 0.01$, and $^{\#\#\#}p < 0.001$ compared with control and $^{*}p < 0.05$, $^{**}p < 0.01$, and $^{***}p < 0.001$ compared with LPS alone.
